# Case Report: Wernicke’s encephalopathy induced by prolonged fasting due to apparent psychogenic dysphagia

**DOI:** 10.3389/fnbeh.2026.1780775

**Published:** 2026-03-10

**Authors:** Logan Mills, Henry Zou, Akram Alnounou, Morgan Smeltzer, Tessa Kravchenko, Theotonius Gomes

**Affiliations:** Western Michigan University Homer Stryker MD School of Medicine, Kalamazoo, MI, United States

**Keywords:** folate deficiency, mammillary bodies, psychogenic dysphagia, thiamine, Wernicke’s encephalopathy

## Abstract

**Background:**

Wernicke’s encephalopathy (WE) is an acute neurologic emergency resulting from thiamine deficiency that can cause irreversible injury if treatment is delayed. Although often associated with alcohol use, WE also occurs in the setting of malnutrition, dysphagia, chronic illness, and malignancy. Dysphagia-related WE is rare and typically linked to impaired oral intake. We report a case of a 26-year-old woman with 3 months of dysphagia and subsequent poor oral intake who developed WE.

**Case report:**

A 26-year-old woman with type 2 diabetes and hypothyroidism presented for confusion, speech incoherence, and gait instability following 3 months of dysphagia. She was hemodynamically stable, and her thyroid, autoimmune, toxic, and infectious workups were negative. Although repeated computed tomography was negative, magnetic resonance imaging showed signal changes in the mammillary bodies, posterior midbrain, and medial thalami, indicating Wernicke’s encephalopathy. Her thiamine, folate, and B12 levels were replaced, and her mentation and coordination improved. A swallow study showed no physiologic dysphagia. She was discharged on oral thiamine supplementation and referred to psychiatry for the presumed psychogenic etiology of her dysphagia, given the distressing life events prior to hospitalization.

**Significance:**

The classic WE triad of confusion, oculomotor abnormalities, and ataxia presents in a minority of patients; hence, atypical presentations may be overlooked. Our patient demonstrated how WE can be induced by nutritional deficiencies secondary to functional or psychogenic dysphagia. Furthermore, this case highlights the importance of prompt clinical intervention via thiamine repletion despite inconclusive initial imaging, as delayed treatment can result in permanent complications.

## Introduction

Wernicke’s encephalopathy (WE) is an acute neurologic emergency caused by thiamine (vitamin B1) deficiency that can lead to irreversible neurologic injury or death if treatment is delayed (Galvin et al., 2010; Habas et al., 2023; Sinha et al., 2019). Current literature reports that the classic triad of confusion, oculomotor abnormalities, and gait ataxia is only present in a minority of patients, leading to underrecognition of atypical presentations (Galvin et al., 2010; Habas et al., 2023; Sinha et al., 2019). Although historically associated with chronic alcohol use, WE can occur in other settings, including malnutrition, dysphagia, bariatric surgery, chronic comorbidities, and malignancy (Habas et al., 2023; Sinha et al., 2019; Manzo et al., 2014; Patterson et al., 2025). Dysphagia-related WE is uncommon, primarily described in cases of esophageal cancer, chemoradiation, or achalasia, where thiamine deficiency results from poor oral intake (Cornea et al., 2022; Fikhman et al., 2011; Zhang et al., 2022). Current guidelines emphasize early empiric parenteral thiamine repletion in high-risk patients, as stores can be depleted within weeks leading to an insidious onset of symptoms (Habas et al., 2023; Sinha et al., 2019; Manzo et al., 2014). We present a 26-year-old woman with 3 months of dysphagia who developed WE.

## Case presentation

A 26-year-old woman with a history of type 2 diabetes, hypothyroidism, and generalized anxiety disorder presented to the emergency department (ED) with acute confusion and incoherent speech, including repetitive and non-sensical statements. History was provided by the patient’s husband, who noted that the patient experienced progressive difficulty swallowing over the previous 3 months, requiring blended food and crushed medications, leading to an unintentional 100-pound weight loss. He also reported 2 weeks of visual difficulties and poor coordination requiring assistance with walking. Of note, the husband reported that the patient’s symptoms had worsened rapidly after multiple family deaths and the death of her dog 3 months prior.

In the emergency department, the patient was found to be hypertensive at 151/86, tachycardic at 128 beats/min, and tachypneic at 20 breaths/min, with normal oxygen saturation in room air. Her labs were notable for mild leukocytosis (13.9 K/μL), hypokalemia (3.2 mEq/L), and elevated C-reactive protein (1 mg/L). Lactic acidosis was present at a level of 4.1 mmol/L. Her thyroid-stimulating hormone, free T4, acetylcholine receptor antibodies, and muscle-specific kinase antibodies were normal. Troponin, serum acetaminophen, salicylate, and ethanol levels were within normal limits, and viral testing (COVID-19, influenza, RSV) was negative. Vitamin B12 was normal, vitamin B1 was elevated to 259.5 nmol/L, and folate was low at <1.6 ng/mL. Non-contrast computed tomography (CT) of the head showed no acute intracranial abnormality. CT angiography of the head and neck and CT venography were negative for arterial or venous occlusion. Chest radiograph revealed no acute cardiopulmonary process. The patient received intravenous fluids and antiemetics. Neurology was consulted and recommended empiric parenteral thiamine, and the patient was admitted for further evaluation and management.

Physical exam revealed a lethargic and confused young woman. Cranial nerve examination showed ophthalmoparesis with impaired lateral left gaze and associated left-beating nystagmus, along with mild impaired vertical upgaze bilaterally. Motor strength was intact with equal reflexes bilaterally, though patellar and Achilles reflexes were absent bilaterally. Coordination was intact. Gait examination was initially deferred due to significant pain.

**Figure 1:**
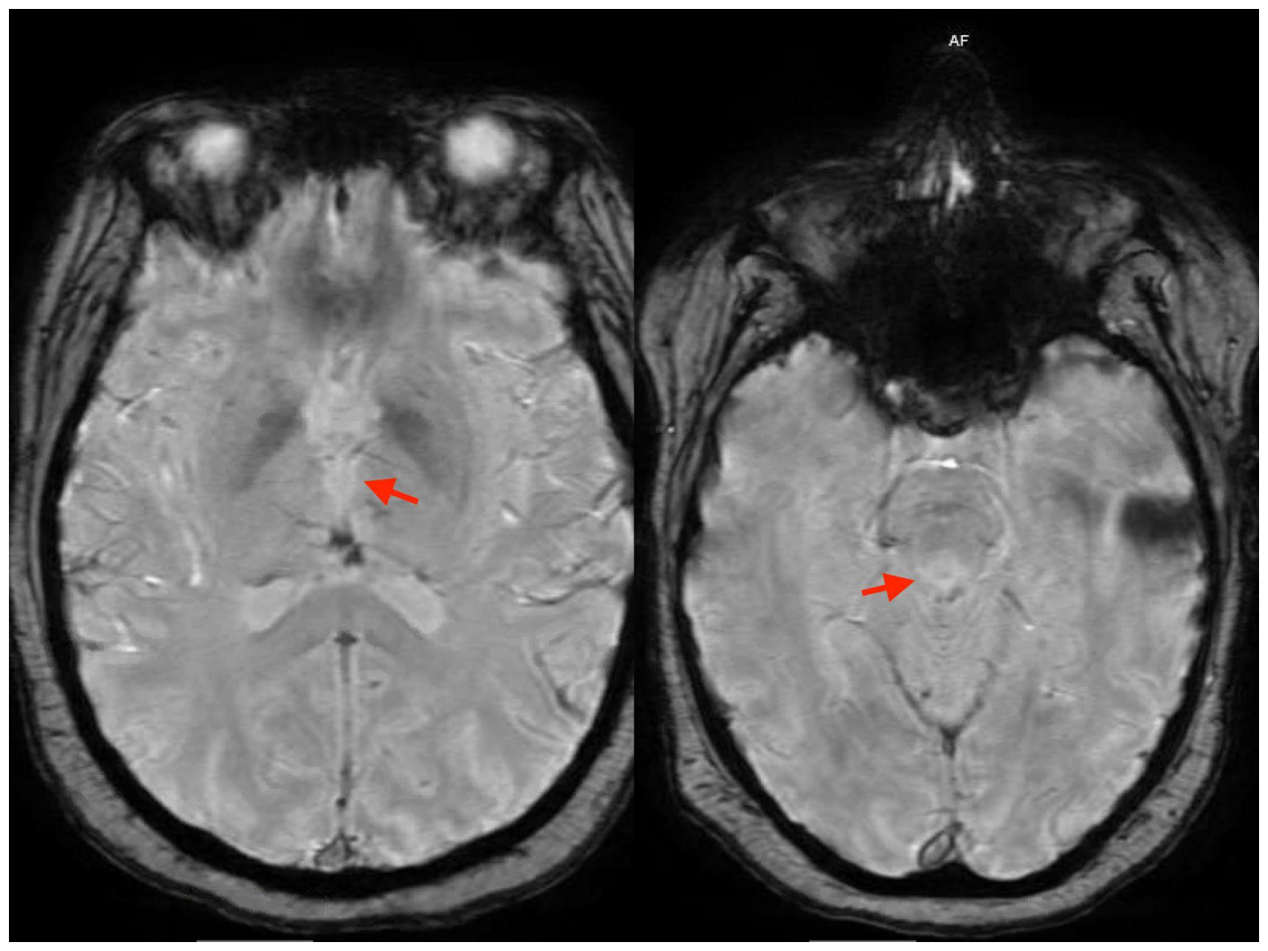
Signal changes in the bilateral medial thalami (left) and mammillary bodies (right) on MRI.

On admission, brain magnetic resonance imaging (MRI) demonstrated signal abnormalities involving the bilateral mammillary bodies, posterior midbrain, and medial thalami consistent with Wernicke’s encephalopathy (Figure 1).

The patient received high-dose intravenous thiamine (500 mg three times daily for 3 days, followed by 250 mg daily for 5 days), along with folate, vitamin B12, and electrolyte repletion. A swallow study conducted by a speech and language pathologist showed no physiologic dysphagia. Her mental status and gait improved during hospitalization. She was discharged home with assistance as needed, a two-wheeled walker, outpatient physical therapy, and oral thiamine 100 mg daily to be continued until outpatient primary care follow-up in 1 week.

**Figure 2:**
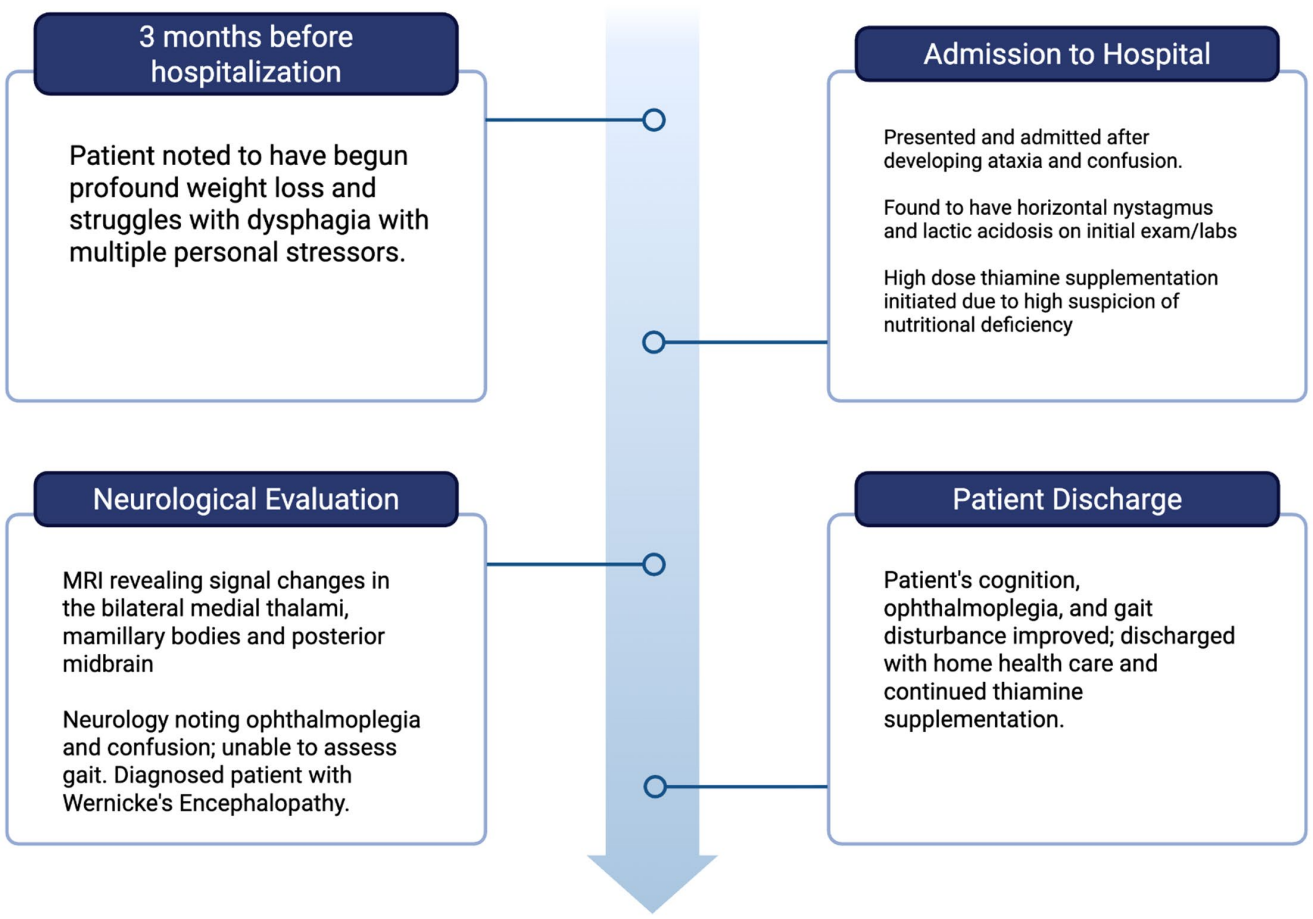
Timeline of the patient’s clinical course. Image created with BioRender.com under an academic license.

At discharge, the most likely differentials for the patient’s dysphagia were functional dysphagia versus the effect of thiamine deficiency itself. There have been documented cases of thiamine deficiency causing dysphagia as an initial presenting symptom, sometimes occurring with as little as 2 weeks of poor intake; therefore, it is possible her appetite loss resulted in dysphagia, leading to her precipitous clinical decline (Cornea et al., 2022; Patterson et al., 2025). Regardless, this dysphagia and malnutrition overall likely led to the patient’s presentation consistent with Wernicke’s encephalopathy. For this reason, the patient was given an outpatient psychiatry referral at discharge; however, no psychiatry visits were documented in her electronic medical record until 1-month post-discharge (Figure 2).

## Patient perspective

The patient describes the weeks leading up to her hospitalization as a “consuming” cycle of depression and anxiety following a choking incident. This caused her to have a severe aversion to eating, which she initially rationalized as dehydration or fatigue, not realizing the severity of her progressive malnutrition. She mentions that it was hard for her to tell how serious her illness was becoming at the moment. The day she presented to the emergency department, she noted almost complete amnesia about her time spent there.

By the time she was transferred to the tertiary care center and initiated on thiamine replacement, she reported a rapid and significant improvement in cognition and a sense of returning close to her “normal self.” Despite her significant improvement in her cognition, she notes that she was still distressed by her continued double vision and inability to move one of her eyes. She mentions that this has continued to improve.

Reflecting on her care, the one thing she emphasized is that she wishes other doctors knew about her situation, which is the importance of thoroughly evaluating and listening to patients. Prior to her ultimate presentation, she had presented to the same emergency department with severe nausea and vomiting and an inability to swallow. At that time, she was treated with antiemetics and did not undergo further diagnostic testing. She is grateful for her subsequent care, but wishes that healthcare professionals would “listen to the patient” and investigate closely when patients are undergoing progressive deterioration rather than attributing symptoms solely to psychiatric causes.

## Discussion

This case describes an uncommon presentation of Wernicke’s encephalopathy (WE) resulting from prolonged dysphagia-induced malnutrition in the setting of significant personal stressors. Previously reported cases of non-alcoholic, dysphagia-induced WE have most often been attributed to structural or iatrogenic causes, including untreated achalasia, esophageal cancer, surgical interventions including laparoscopic sleeve gastrectomy, and other conditions associated with impaired intake or absorption (Zhang et al., 2022; Huq et al., 2025; Rodriguez et al., 2023; Pardo-Aranda et al., 2016). In contrast, our patient’s dysphagia was presumed to be psychogenic, supported by the patient’s profound weight loss in response to numerous personal stressors, likely manifesting as an adjustment disorder with severe features of appetite loss. She may have suffered from psychogenic dysphagia compounded by thiamine deficiency, or the condition could have been primarily thiamine-induced, triggering a positive feedback loop of nutritional depletion and worsening symptoms. This case illustrates the importance of maintaining a high index of suspicion for WE in patients with inadequate oral intake of any etiology, particularly given that non-alcoholic WE is more likely to present with atypical features and a subacute onset (Galvin et al., 2010).

**Figure 3:**
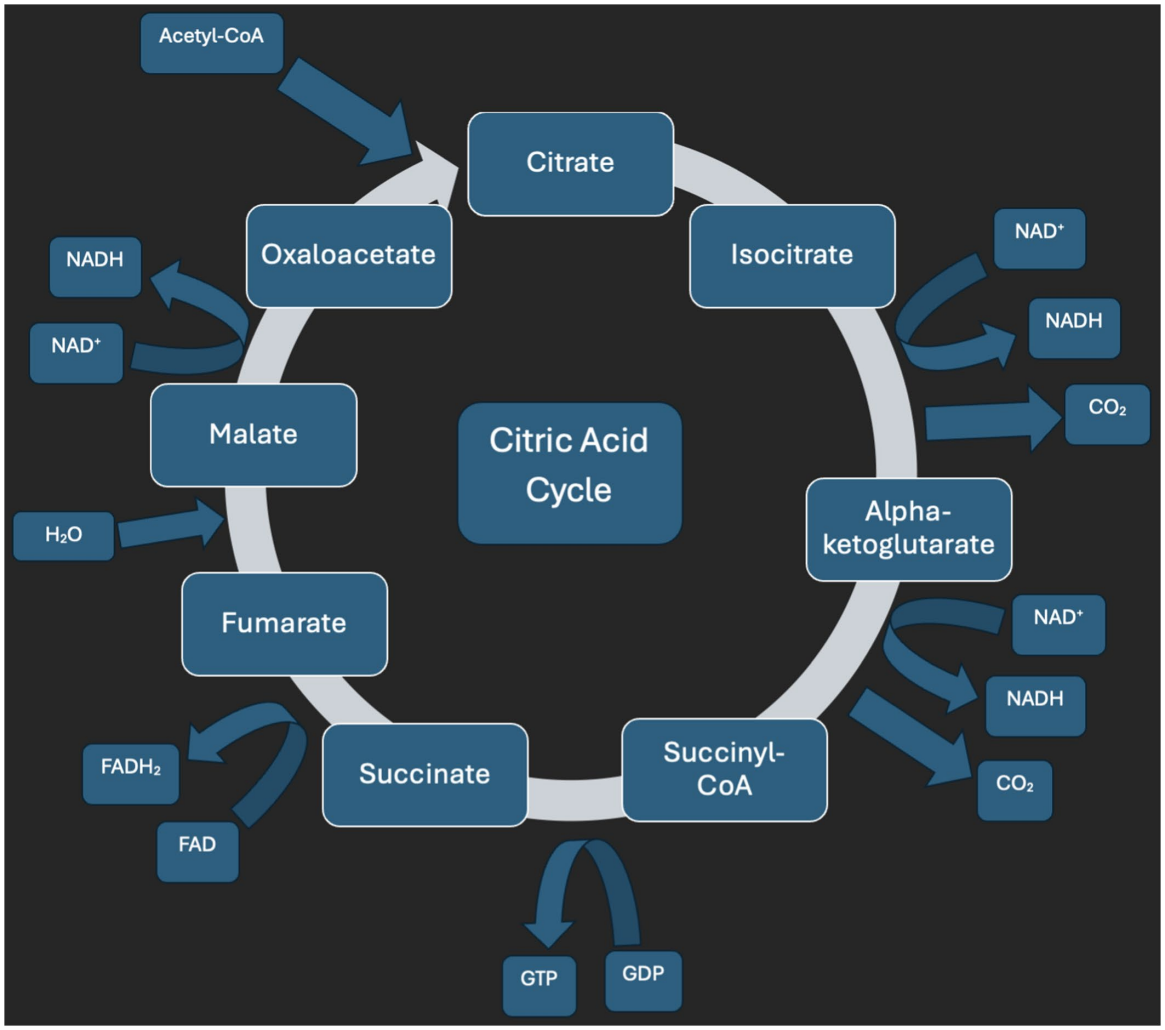
Citric acid cycle diagram. Image created with Microsoft Word under an academic license.

Severe thiamine deficiency is a condition with potentially high morbidity and mortality, making early identification and prompt management crucial. Thiamine is an essential cofactor in multiple metabolic pathways, including glucose oxidation via the Embden–Meyerhof pathway, the citric acid cycle through alpha-ketoglutarate dehydrogenase activity (Figure 3), and the pentose phosphate pathway (Lonsdale, 2006).

In thiamine deficiency, impaired oxidative metabolism leads to neuronal death, especially in the more vulnerable regions of the nervous system, including the midline mesencephalon, cerebellar vermis, and peripheral nerves (Fikhman et al., 2011). These pathophysiologic changes result in the classic triad of confusion, ataxia, and ophthalmoplegia; however, this triad is present in only 8% of confirmed WE cases (Galvin et al., 2010). Atypical manifestations are common, so heightened clinical suspicion is essential, given any concern about reduced nutritional intake, even in patients without a history of alcohol use or other traditionally associated conditions. Prompt initiation of high-dose thiamine supplementation often rapidly reverses symptoms, as seen in our case. Delay in thiamine administration or inadequate replacement can result in progression to Korsakoff syndrome, resulting in permanent neuropsychiatric changes (Chandrakumar et al., 2018).

WE is primarily a clinical diagnosis, as no laboratory test or imaging study is independently diagnostic of the condition. Evidence of thiamine deficiency may be supported by laboratory findings, but results should not delay treatment when suspicion is high. Measurement of erythrocyte transketolase activity (EKTA) before and after thiamine repletion can suggest thiamine deficiency if baseline activity is low and improves following supplementation, though this test is often not readily available (Chandrakumar et al., 2018). Whole blood thiamine levels can also be measured, but normal values do not exclude deficiency, and empiric thiamine administration should not be delayed while awaiting these results.

Neuroimaging can also support the diagnosis of WE. The most common findings on magnetic resonance imaging (MRI) in patients with WE are bilateral hyperintensities involving the mammillary bodies, thalamic nuclei, periventricular gray matter, colliculi, and cerebellum (Sullivan and Pfefferbaum, 2009). While T2-weighted MR imaging may reveal these changes, fluid-attenuated inversion recovery (FLAIR) images are preferred, as this sequence better demonstrates hyperintensities in edematous brain tissue. However, MRI sensitivity is limited, and a normal MRI does not exclude WE; therefore, prompt recognition of associated symptoms is essential, regardless of imaging findings. In this case, early identification of WE allowed for prompt thiamine administration, likely contributing to the patient’s favorable neurologic outcome. Overall, laboratory testing and imaging are not required for diagnosis and should not delay treatment in patients with suspected WE and nutritional deficiency (Habas et al., 2023).

In our patient, WE was attributed to likely psychogenic dysphagia, also called medically unexplained oropharyngeal dysphagia (MUNOD), which is considered a diagnosis of exclusion (Verdonschot et al., 2019). In a study of 14 MUNOD patients, six (42.8%) tested positive for clinically significant anxiety and depression; however, they did not score higher on the dysphagia severity scale relative to MUNOD patients without affective disorders (Verdonschot et al., 2019). Psychogenic dysphagia may induce WE through weeks to months of inadequate thiamine intake, but dysphagia may also be an atypical presenting symptom of WE that precedes other classic neurological deficits (Cornea et al., 2022). Given that our patient reported 3 months of dysphagia after experiencing grief and loss and demonstrated no physiologic dysphagia in her inpatient swallow study, there was high suspicion for psychogenic dysphagia as the etiology for her WE.

The cornerstone of Wernicke’s encephalopathy management is thiamine supplementation administered immediately upon suspicion of WE, as delay in replacement can result in progression to Korsakoff syndrome and permanent neuropsychiatric changes (Rodriguez et al., 2023; Chandrakumar et al., 2018). Current recommendations favor parenteral high-dose thiamine for optimal bioavailability in severe deficiency (Galvin et al., 2010). In our patient, early administration of intravenous thiamine resulted in significant clinical improvement within days of hospitalization, consistent with outcomes in other reported cases of dysphagia-induced WE (Cornea et al., 2022; Fikhman et al., 2011; Zhang et al., 2022). This case underscores the importance of empiric thiamine therapy in at-risk patients presenting with neurologic symptoms and poor nutritional intake, regardless of the underlying cause.

## Conclusion

Contemporary literature points to a wide range of etiologies of thiamine deficiency leading to Wernicke’s encephalopathy (WE). The presentation of the disease can vary dramatically and, more often than not, will not exhibit the classic triad of ataxia, ophthalmoplegia, and confusion. This particular case demonstrates that psychiatric illness and suspected functional disorders of swallowing and malnutrition should prompt high suspicion of nutritional deficiencies, especially when accompanied by comorbid symptoms. Early intervention in WE is critical, as there is minimal risk to aggressive empiric thiamine repletion, but significant potential morbidity from delayed treatment.

## Data Availability

The original contributions presented in the study are included in the article/supplementary material, further inquiries can be directed to the corresponding author/s.
